# Lessons Learned from the Implementation of a Person-Centred Digital Health Platform in Cancer Care

**DOI:** 10.3390/curroncol29100564

**Published:** 2022-09-29

**Authors:** Saima Ahmed, Karine LePage, Renata Benc, Guy Erez, Alon Litvin, Annie Werbitt, Gabrielle Chartier, Carly Berlin, Carmen G. Loiselle

**Affiliations:** 1 Division of Experimental Medicine, Faculty of Medicine and Health Sciences McGill University, Montreal, QC H4A 3J1, Canada; 2 Centre Intégré Universitaire de Santé et de Services Sociaux (CIUSSS) du Centre-Ouest-de l’Île-de Montréal, Montreal, QC H3T 1E2, Canada; 3 Belong.life Inc., New York, NY 10001, USA; 4 Hope & Cope, Montreal, QC H3T 1E2, Canada; 5 Department of Oncology, Faculty of Medicine and Health Sciences, McGill University, Montreal, QC H4A 3T2, Canada; 6 Ingram School of Nursing, Faculty of Medicine and Health Sciences, McGill University, Montreal, QC H3A 2M7, Canada

**Keywords:** digital health, oncology, eHealth, mHealth, implementation, education

## Abstract

The SARS-CoV-2 (COVID-19) pandemic has accelerated the development and use of digital health platforms to support individuals with health-related challenges. This is even more frequent in the field of cancer care as the global burden of the disease continues to increase every year. However, optimal implementation of these platforms into the clinical setting requires careful planning and collaboration. An implementation project was launched between the Centre intégré universitaire de santé et de services sociaux (CIUSSS) du Centre-Ouest-de-I’Île-de-Montreal and BELONG—Beating Cancer Together—a person-centred cancer navigation and support digital health platform. The goal of the project was to implement content and features specific to the CIUSSS, to be made available exclusively for individuals with cancer (and their caregivers) treated at the institution. Guided by Structural Model of Interprofessional Collaboration, we report on implementation processes involving diverse stakeholders including clinicians, hospital administrators, researchers and local community/patient representatives. Lessons learned include earlier identification of shared goals and clear expectations, more consistent reliance on virtual means to communicate among all involved, and patient/caregiver involvement in each step to ensure informed and shared decision making.

## 1. Introduction

Encompassing a broad-spectrum of health technologies from mobile health apps (mHealth), health information technology, wearable devices, telehealth, telemedicine, to personalized medicine; digital health (DH) has the potential to dramatically enhance care provision and quality through a more streamlined and personalized approach [1,2,3]. DH platforms have been linked to enhanced access to health and social services, contained costs, and more sustained patient follow-ups [4,5,6,7].

DH platforms are increasingly popular among individuals with cancer as they often contain large repositories of content, information exchange channels, and support for patients’ (changing) cancer-related needs [8,9,10,11,12] The National Cancer Institute’s Health Information National Trends Survey (HINTS) has documented the popularity of individuals with cancer using the internet to access cancer-related information increasing from 49.5% in 2003 to 76.9% in 2017 [13]. DH can enhance care that is person-centred from diagnosis to treatment through survivorship or palliative care and caregiver bereavement, empowering and supporting through the promotion of health literacy, shared decision-making, and illness self-management [4,14,15].

The World Health Organization’s global strategies on people-centred health services and digital health outline the integration of different stakeholders as essential to strengthening the implementation of innovation—leveraging and maximizing diverse knowledge and experiences, allowing potential challenges to be addressed jointly, accelerating the development of integrated health services, and advancing towards the United Nations Sustainable Development Goals [2,16]. Collaboration ensures the potential for best results; patients and caregivers as the end-users can share their needs, preferences, and experiences, while healthcare professionals each bring their expertise and proficiency [17,18]. Lastly, including platform developers throughout the implementation process allows the reality of what is technologically possible to be gauged. In sum, engaging multi-stakeholders in DH is beneficial for all involved [19]. However, despite the growing evidence of the importance of patients as key stakeholders, to date, their involvement in DH research and implementation remains limited [20]. Literature supports that their early and continuous engagement as stakeholders is essential, increasing the methodological quality of work and fostering collective intelligence [20].

Multi-stakeholder collaboration is generally understood to be the “integration of activities and knowledge that requires a partnership of shared authority and responsibility” [21]. Evidence supports that interprofessional collaboration in healthcare enhances patient care with collaborative teams being higher functioning; providing care that is well-coordinated and person-centred [22,23]. In this article, we report on and explore multi-stakeholder collaboration in the implementation of a DH platform in cancer care; our objective is to provide a better understanding of and help guide future projects by highlighting the facilitators and challenges faced.

In 2018, the Centre intégré universitaire de santé et de services sociaux (CIUSSS) du Centre-Ouest-de-I’Île-de-Montreal—an integrated university health and social services centre located in Montreal, QC, Canada—put forth a new mandate to transform the way care is delivered; providing “care wherever the patient is” and promoting an optimal experience with the healthcare system by employing a strategy of “aggressive digital health transformation” [24,25]. Especially pertinent considering the new models of remote and hybrid care deployed during the SARS-CoV-2 (COVID-19) pandemic, as well as considering the higher risk for virus-related complications immunocompromised individuals with cancer face, the CIUSSS’s Segal Cancer Centre sought to increase access to supportive resources. Building upon the promising results of a pilot study among women with gynecological cancer using the DH platform, BELONG—Beating Cancer Together (https://cancer.belong.life/; accessed on 20 March 2020)—considered “the world’s largest social network for individuals with cancer, caregivers and healthcare professionals”, a collaborative project between the CIUSSS and BELONG was launched in 2021 [26]. The goal of the project was to implement content and features specific to the CIUSSS on BELONG, made available exclusively for individuals with cancer and their caregivers at the institution. To meet these goals, an interdisciplinary team of diverse stakeholders was established and incorporated at various stages of the five-step implementation process as outlined in Table 1, these stakeholders included Hope & Cope—a local community volunteer-based non-profit organization—as well as patient representatives, clinicians, hospital administrators, and researchers. Step 1 began the project by bringing together all stakeholders at the CIUSSS. Step 2 introduced a core group of stakeholders from the CIUSSS to a core group from BELONG. Step 3 was the combined and continued efforts between these core groups for over a year. Step 4 examined the impressions of platform from the perspectives of CIUSSS and community stakeholders through informal focus groups. Step 5 launched the platform, introducing additional stakeholders in the process of clinical implementation.

Collaboration among these stakeholders and the BELONG development team was present from project onset, through implementation and operationalization; the diverse expertise of each was integrated to ensure the best possible results. Whereas several frameworks related to collaboration in healthcare have been put forward, D’Amour’s [27] Structural Model of Interprofessional Collaboration was chosen due to its theoretical roots, evidence-based nature and extensive use [28].

## 2. Structural Model of Interprofessional Collaboration

**Figure 1:**
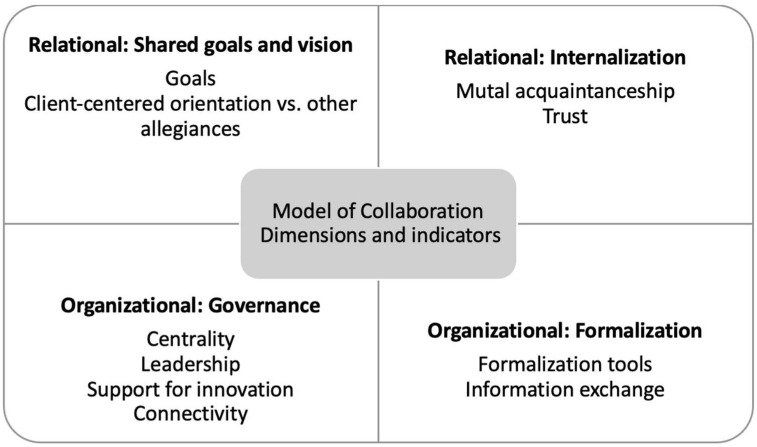
The four dimensions and ten indicators of the Structural Model of Interprofessional Collaboration [29].

As seen in Figure 1, the model has four dimensions: the first two, (1) shared goals and vision, and (2) internalization, focus on “relationships between individuals”, while the others, (3) formalization and (4) governance, touch upon how these relationships interact at the organizational level [29]. Each dimension is further classified into indicators that determine the level of collaboration, from level 1 to 3: level 1 is the lowest as potential or latent and not yet existing, level 2 is developing and thus not yet established, and level 3 as active and stable and the highest level of collaboration [29].

## 3. Intersection of Relational and Organizational Collaboration

All stakeholders shared an allegiance to creating a meaningful final product, thus being client-centred. To the BELONG development team, their client was the CIUSSS, supporting the institution in engaging with their cancer population. However, to the patient representatives, non-profit organization, clinicians, hospital administrators, and researchers, their client was to the patient population itself, promoting optimal experiences along the cancer care journey through use of the platform.

Recognizing and agreeing upon shared common goals is an indispensable starting point for collaboration. In our case, the project began with a kickoff meeting with institutional stakeholders at the CIUSSS where interdisciplinary strategies were discussed, objectives and priorities for the project were defined. It was mutually understood and agreed upon that the explicit goal was to create a private closed community in BELONG for patients and caregivers at the institution. Thus, it would be necessary to modify already-existing features and include additional content to make the platform specific to the organizational context, to be available in English and French. Upon meeting with the BELONG development team, the goal was further conceptualized to include: (1) Patients Area, a supportive space connecting individuals with others living with cancer, sharing concerns, knowledge, strategies, and general posts from trained volunteers; (2) Caregivers Area, a supportive space connecting people caring for someone living with cancer, sharing concerns, knowledge, strategies, and general information from trained volunteers; and (3) a folder in “My files”, providing educational information, videos and documents, as well as relevant resources from reliable government, university, hospital and non-profit charitable organization websites.

The biggest challenge encountered was that clear parameters and expectations surrounding the logistics of the implementation process were not discussed early on among and between the CIUSSS core group and the BELONG development team. This led to small misunderstandings surrounding project scope and development in the early phase. For instance, role clarity—i.e., who was responsible for which task, such as translation from English to French—was not explicit. In addition, as the platform was new to many, they did not initially understand the scope of all its functionalities and limitations.

Fortunately, transparent communication with a clear and constant focus on the overarching goal of the project helped redirect efforts over time. All stakeholders involved shared relevant information regarding the project on a consistent basis through emails and virtual meetings. Despite being in different locations and time zones, a core group forming the BELONG project and technical support based in Israel, along with nursing management and a doctoral researcher, located in Montreal, met weekly for one year over Zoom. This provided both formal and informal opportunities to interact on a personal and professional level. Despite busy schedules, finding a time to meet consistently helped build mutual acquaintanceship. These meetings were especially important in exchanging information about the status of the project, new tasks to be completed, as well as virtual follow-up from what had been discussed in written exchanges over the week. As clear and tangible expectations and responsibilities were established and agreed upon, the project steadily progressed.

In step 3, the three aspects of the project that were conceptualized earlier were operationalized (Table 2). Through the process of consultation with community stakeholders, it was determined that both Patient and Caregiver Area’s would be virtual communities, under the supervision of trained volunteers from Hope & Cope. Individuals using the platform could post questions or share experiences, others may respond through “hugging” or commenting. In addition, the Support Program Manager would answer questions and share relevant information such as upcoming events, as well as information on wellness programs and peer support resources.

The Files area would be a “one-stop shop”, a comprehensive digital space where patients and caregivers could find what they need, conveniently, in a dedicated space. The content was based on input provided by nurses, patient, and caregiver partners, and subsequently reviewed by the same stakeholders prior to implementation. The content includes information that was previously only taught in-person at the beginning of the cancer trajectory. Having this information integrated into the BELONG platform reinforces teaching performed by oncology nurses and enhances access to informational support at various phases of the patient cancer experience. In addition, community resources and links to reliable websites are also provided. Most of these resources were already being recommended by different care teams at the cancer center. Stakeholder feedback indicated that these resources are useful and of interest however, not widely advertised, or confusing/overwhelming to find online. Integrating BELONG into the care trajectory to complement already existing resources was a strong motivator for collaboration among stakeholders, as they understood its potential to contribute further to optimal care and sustainably alleviate strain on the healthcare system.

**Figure 2:**
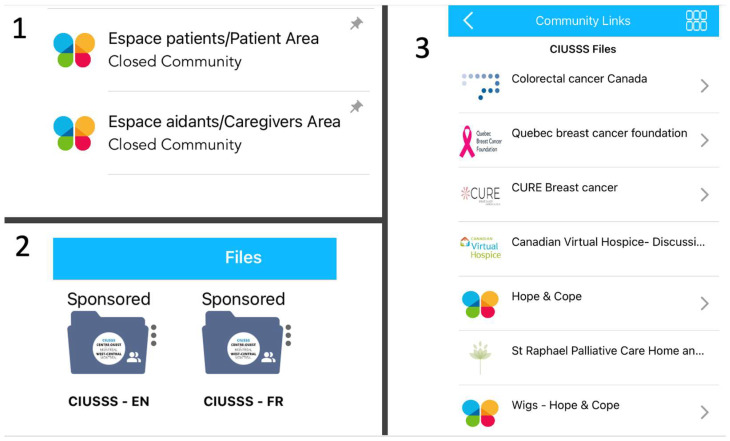
Content and features of the BELONG App for patients and caregivers closed community (1) and personal health files/resources (2 and 3).

Once a working version of the closed community was ready, all stakeholders were invited to review the platform; this included patient partners as well as clinicians and community representatives. They were thereafter invited to take part in a virtual focus group where they provided feedback on the look, features, and ease of use that was used for finetuning. As many stakeholders had been previously consulted for content itself, most of the feedback given related to the look of the Files area; wanting it to be easier to find and with more images to complement the text content. Once feedback was gathered, BELONG’s technical team made suggestions on potential venues to accommodate these requests, core stakeholders discussed and decided how to proceed, with the CIUSSS team providing local context. As seen in Figure 2, the CIUSSS Files were spotlighted for each CIUSSS user as “Sponsored”, highlighting and promoting these Files in BELONG by separating them from all other features and consistently being at the top of the page. In addition, an image corresponding to the organization providing each resource and/or link was added. This feedback served as a reminder to the core team who developed content that first impressions, visuals, and how the content is presented should not be underestimated.

One central authority at the institution, a clinical-administrative nursing coordinator overseeing the project, was a key player at every step, from helping establish the collaboration, to holding accountability for work and inviting relevant stakeholders. The project manager at BELONG played a significant role in coordinating and communicating the needs of the institution to the development and technical team. Emergent leadership roles taken on by BELONG technical support and a Clinical Nurse Consultant at the institution were essential in moving the project forward. These partnerships ensured that the expertise of every individual was utilized and met the shared goal for the best possible outcome, therefore fostering collaboration as decision-making power was shared, rather than fragmented.

## 4. Outcomes

The CIUSSS closed community in the BELONG platform was launched in February 2022, delayed by many months due to the third wave of COVID-19. Prior to the launch, additional stakeholders were bought on to develop and execute a clinical implementation plan that would integrate BELONG into the cancer care trajectory at the CIUSSS, engaging with patients, caregivers, families, healthcare professionals and hospital staff. This plan included communication pieces such as newsletter articles and blog posts, printed posters, video recorded presentations and trainings on how to download and access the platform developed by the Master’s nursing students, as well as presentations at tumor boards and staff team meetings. Patient and caregiver partners were once again consulted at this step, specifically in the development of promotional materials such as posters (e.g., what colors would catch their eye, wording, etc.).

For the first two weeks following the launch, the CIUSSS core team joined by the clinical implementation team were physically present in the oncology outpatient clinic and outpatient chemotherapy unit, interacting with patients and healthcare providers by providing in-person demonstrations of BELONG and its features. Those who showed interest in downloading and using the platform were asked if they would like to provide feedback on their experience. If they agreed, they were emailed a questionnaire after a month of platform use. Questionnaires were for program quality improvement only (not aimed at answering a research question) and received approval from the Quality Department of the institution prior to being sent out. In total, we received 32 responses: 16 from patients and 16 from health care providers. As summarized in Table 3, feedback from patients indicated that the platform was easy to use (43% strongly agree; 57% agree) and had the potential to help with side effects of treatment (25% strongly agree; 12.5% agree; 50% somewhat agree), and a little more than half of the respondents felt that it had prevented Emergency Room visits (57% yes). As summarized in Table 4, healthcare providers found the in-person and virtual trainings prepared them adequately to support patients using the platform (31% strongly agree; 62% agree) and saw the platform as a potentially helpful tool for patients (50% strongly agree; 37.5% agree; 12.5% somewhat agree).

The hands-on approach at launch was successful, as close to 100 new users joined the platform in the first two weeks. Subsequently, the closed community averaged three new users per week. To date, the most frequently accessed content determined by the total number of views in the Files area are (1) Educational Videos, (2) Getting Started, (3) Community Links, (4) Resources and Websites, and (5) Symptom Management, demonstrating that the platform is helping meet these informational needs.

## 5. Recommendations

Integrating stakeholders’ knowledge, feedback and expertise/experience ensured relevance of the platform for patients and caregivers at the institution, providing a supportive community for individuals to connect with others living with/or caring for someone with cancer, receive information and access useful resources.

Herein, the Structural Model of Interprofessional Collaboration [27] provided a robust and practical framework, allowing for deeper exploration and analysis of this complex project. The essential elements of collaboration included shared goals among all stakeholders, compelling everyone to work together, coordinating their efforts over time to achieve results that were satisfactory to all. From the outset, locally assigned leadership set a clear and explicit direction, reinforced over time through communication channels and information exchange. As stakeholders interacted and communicated with each other, they began to better understand shared responsibilities and expectations, building collective trust. Transparency and respect were key facilitators to communication as individuals with varied backgrounds and experiences were integrated as stakeholders. In contrast, scheduling was at times a barrier, perhaps to be expected when bringing together such diverse groups. Recommendations to overcome this include reliance on common information exchange channels and planning tools—formalization—allows for communication among stakeholders in a rapid and comprehensive manner. A subsequent partnership developed between emergent leaders within each organization, including individuals who took on the responsibility to meet the needs of the project as they emerged and sharing their expertise within the team. For instance, the doctoral researcher with experience using the platform in a previous pilot study provided guidance to others at the CIUSSS regarding platform features, use, and uptake. While the BELONG team handled technical aspects of the platform, they also shared lessons on what they had learned from past projects. The team at the institution kept the project and decisions grounded within clinical realities of their setting and patient population. When needed, other professionals were bought in, such as administrators, additional clinicians, and patient representatives. Based on D’Amour’s [29] typology and indicators, this project can be described as having had substantially active collaborations, described as level 3.

## 6. Conclusions

As healthcare moves towards a more holistic approach to person-centred care [10], the respectful, responsive, and tailored means to patient and caregiver needs can be facilitated through digital health platforms. We have underscored the importance of multi-stakeholder collaboration to ensure person-centred design that is grounded in clinical/disease realities and inclusive to the pressing needs of users. Whereas the COVID-19 pandemic accelerated the uptake of digital technology in cancer care settings, it also came with implementation challenges. We must now work on sustained post-pandemic solutions that meet the (changing) needs of users. Whereas increasing awareness of patients and caregivers of the most relevant supportive platforms remains a challenge, in-person demonstrations have shown to be the most successful. However, resource availability such as time and personnel remains a limiting factor. To address this, trained volunteers from Hope & Cope can successfully orient patients and caregivers to the BELONG features in waiting rooms.

Next steps for our team include increasing platform uptake by further integration into community and clinical practice, as well as the addition of supportive resources specific to cancer types and treatments. Future projects should continue to address potential challenges through early identification and explicit discussion of these as well as shared goals, expectations, and collective solutions across planning, conceptualization, content and feature integration, implementation, and evaluation.

## Figures and Tables

**Table 1:** Overview of implementation process—steps, stakeholders, and outcomes.

Steps	1	2	3	4	5
Description	Project kickoff meetings at CIUSSS	Initial meeting with CIUSSS and BELONG	Virtual weekly meetings for one year	Focus Groups	Launch of Platform
Stakeholders involved	Oncologist, nurses, administrators, researchers, local community non-profit organization, patient and caregiver partners	BELONG core group: project manager, sales, and technical support	BELONG core group	Nurses, patient and caregiver partners, local community non-profit organization	Patient and caregiver partners, clinicians and administrators, CIUSSS core group, local community non-profit organization
		CIUSSS core group: Clinical nurse consultant, nursing administration, doctoral researcher, Master’s students	CIUSSS core group		
Outcomes	Priorities and objectives for project defined	Conceptualization of CIUSSS closed community in BELONG	Integration of CIUSSS content into closed community: Patient and caregiver areas and “My Files”	Finetuning of closed community based on feedback	Clinical implementation, onboarding of patients and families

**Table 2:** Overview of features available in BELONG for the CIUSSS closed community.

Feature	Type of Support	Content	Details
Patient Area	Peer	Virtual Community	Post: question or share experience “Hug” or comment on a post Details on upcoming community events Wellness and peer support
Caregivers Area	Peer	Virtual Community	Post: question or share experience “Hug” or comment on a post Details on upcoming community events Wellness and peer support
CIUSSS Files	Informational	Booklets, websites, resources	Community Links Educational Videos Febrile Neutropenia Getting Started Intro to Segal Cancer Centre Palliative Care Resources and Websites Resources JGH Special Populations Symptom Management Transportation and Financial Aid When to call your team ASAP Your tools

**Table 3:** Overview of patient provider quality improvement feedback on experience using platform.

Patient Report (*n* = 16)	Strongly Agree *n* (%)	Agree *n* (%)	Somewhat Agree *n* (%)	Neither Agree nor Disagree *n* (%)	Yes *n* (%)	No *n* (%)
The App was easy to use	7 (43)	9 (57)	0 (0)	0 (0)	n/a	n/a
The information in the App allowed me to take care of my side effects related to my treatments.	4 (25)	2 (12.5)	8 (25)	2 (12.5)	n/a	n/a
Using the information in the App prevented a visit to the Emergency Room	n/a	n/a	n/a	n/a	9 (57)	7 (43)

**Table 4:** Overview healthcare provider quality improvement feedback on platform and training provided.

Healthcare Provider Report (*n* = 16)	Strongly Agree *n* (%)	Agree *n* (%)	Somewhat Agree *n* (%)	Neither Agree nor Disagree *n* (%)
The training session prepared me to support patients using the App.	5 (31)	10 (62)	0 (0)	1 (7)
This App may help my patients manage their health.	8 (50)	6 (37.5)	2 (12.5)	0 (0)
